# Encapsulation of Biosynthesized Nanosilver in Silica Composites for Sustainable Antimicrobial Functionality

**DOI:** 10.1002/gch2.201800048

**Published:** 2018-08-19

**Authors:** Khaled S. Abou‐El‐Sherbini, Mohey H. A. Amer, Mohamed S. Abdel‐Aziz, Esmat M. A. Hamzawy, Walid Sharmoukh, Mohamed M. Elnagar

**Affiliations:** ^1^ Department of Inorganic Chemistry National Research Centre 33 El Bohouth St. (former Tahrir St.) 12622 Dokki Giza Egypt; ^2^ Higher Institute of Engineering and Technology kilo 112 Cairo Alex Agricultural Road Tanta 31739 Egypt; ^3^ Department of Microbial Chemistry National Research Centre 33 El Bohouth St. (former Tahrir St.) 12622 Dokki Giza Egypt; ^4^ Department of Glass National Research Centre 33 El Bohouth St. (former Tahrir St.) 12622 Dokki Giza Egypt

**Keywords:** antimicrobial activity, dispersion, embedment, silver nanoparticles, stability

## Abstract

Silver nanoparticles (AgNPs) have become known as a broad‐spectrum antimicrobial agent. The antimicrobial activity of AgNPs is dependent on the particle size and the dispersion status. In this study, a simple and effective approach is developed for sequestering the biosynthesized AgNPs in silica composites during the gel formation of MCM‐41. Composites with different Ag concentrations of 0.034% (Ag1@MCM‐41), 0.151% (Ag2@MCM‐41), and 0.369% (Ag3@MCM‐41) are synthesized and then heated at 400 °C to produce Ag1@MCM‐41H, Ag2@MCM‐41H, and Ag3@MCM‐41H, respectively. The samples are characterized by flame atomic absorption spectrometry, Fourier‐transform infrared spectroscopy, X‐ray diffraction, N_2_ physisorption, scanning electron microscopy, transmission electron microscopy, and thermogravimetric analysis. The AgNPs are confirmed to be highly dispersed in the amorphous silica framework. The antimicrobial activity of the AgNP–silica samples is investigated against *Staphylococcus aureus*, *Escherichia coli*, and *Candida albicans* using the cup–plate and the plate‐count techniques. The results show an excellent antimicrobial effect of these samples against the studied microorganisms. Importantly, the AgNP–silica samples are found to be stable up to 58 months under ambient conditions. These stable and powerful antimicrobial composites provide a more practical and effective strategy for combating biomedical pathogens and public health threats.

## 1 Introduction

Silver nanoparticles (AgNPs) have received the greatest attention among the noble metallic NPs because of their distinctive physicochemical and biological properties. They have been utilized in several fields, including water purification, H_2_ production from amminetrihydridoboron, biomedicine, cosmetics, membrane filtration (fouling control), and food preservation.^1^, ^2^, ^3^, ^4^, ^5^ Considering the antimicrobial activity of Ag, AgNPs are better than other Ag forms even when applied at lower concentrations.^6^, ^7^ The unique properties of nanoscale Ag, including the high specific surface area and high fraction of surface atoms, facilitate the fatal contact with microorganisms.^8^, ^9^ Moreover, AgNPs have little chance of eliciting drug resistance, which is an urgent issue for many antibiotics.^10^, ^11^ Thus, AgNPs have emerged as effective antimicrobial agents to combat a broad spectrum of pathogenic microorganisms.^12^

The mechanism of the antimicrobial action of AgNPs has not been fully clarified.^13^ It has been suggested that mechanisms can arise from i) Ag ions interact with the sulfhydryl groups of enzymes, causing detrimental structural changes in cellular organelles,^14^, ^15^ ii) NPs may attach to the surface of the cell membrane and disturb its functionality,^13^, ^16^ and iii) NPs generate reactive oxygen species (ROS) that may damage both proteins and DNA.^17^

The concept of sustainable nanotechnology involves the nanoscale control of matter synthesis without effects that may create serious environmental issues. Consequently, the search for more effective synthetic approaches that use less chemicals, organic solvents, and energy has gained significant attention. The environment‐friendly and facile synthesis of NPs has been moving toward the recruitment of biogenic matters, such as different microorganisms, enzymes, and plant extracts that facilitate the reaction. In this regard, many biological sources, including blackberries, spent mushroom substrates, pomegranates, and *Phoenix dactylifera* leaves have been utilized in the synthesis of metal NPs.^18^, ^19^, ^20^ Furthermore, microorganisms such as *Variovorax guangxiensis*, *Sinomonas mesophila*, *Aspergillus fumigatu*, and *Trichoderma asperellum* have been used for the green synthesis of AgNPs owing to their ease of manipulation.^21^, ^22^, ^23^, ^24^ These previous studies have demonstrated the importance of eco‐friendly and benign approaches for rapid, cost‐effective, and safe synthesis of NPs compared with chemical reduction methods.

The physicochemical characteristics of AgNPs, such as particle size, morphology, dispersion, and stability are vital factors of concern for achieving high antimicrobial performance.^25^ AgNPs tend to aggregate in suspensions as a result of their high surface energy, such that their antimicrobial activity sharply decreases over time.^26^, ^27^ Additionally, practical applications of free AgNPs are still limited due to the ultimate health risks of their dissipation into the environment.^28^, ^29^ These drawbacks can be addressed by stabilizing AgNPs in various solid supports. Therefore, the search for suitable supporting matrices that maintain the antimicrobial activity of low NP concentrations and enable rapid NP release remains a key challenge in this field.^30^, ^31^, ^32^ Several supports, such as titanium dioxide,^33^ activated carbon,^34^ and montmorillonite,^35^ have been investigated as Ag‐carrying antimicrobial agents. However, the several drawbacks of these composites, such as their unfavorable biocompatibility and low dispersity, limit their practical applications.^36^, ^37^, ^38^ These limitations result in the continued search for more practical supports with suitable structures.

Silica‐based materials have been preferred for antimicrobial applications due to their hydrophilic nature and biocompatibility.^39^, ^40^, ^41^, ^42^, ^43^, ^44^ Among them, MCM‐41‐type mesoporous silica materials have gained great interest over recent years, mainly due to their unique features, such as, uniform porous structure, large surface area, and excellent chemical and thermal stability.^45^, ^46^ As AgNPs can be immobilized into MCM‐41 to form Ag‐containing materials, these materials are predicted to have more antimicrobial activity than conventional AgNPs.^47^

With these in mind, we report a facile, economical and effective route for the direct sequestration of biosynthesized AgNPs on MCM‐41‐based composites to produce antimicrobial materials with potential sustainability and high efficiency.

## 2 Results and Discussion

### 2.1 Characterization

#### 2.1.1 X‐Ray Diffraction (XRD)

**Figure 1:**
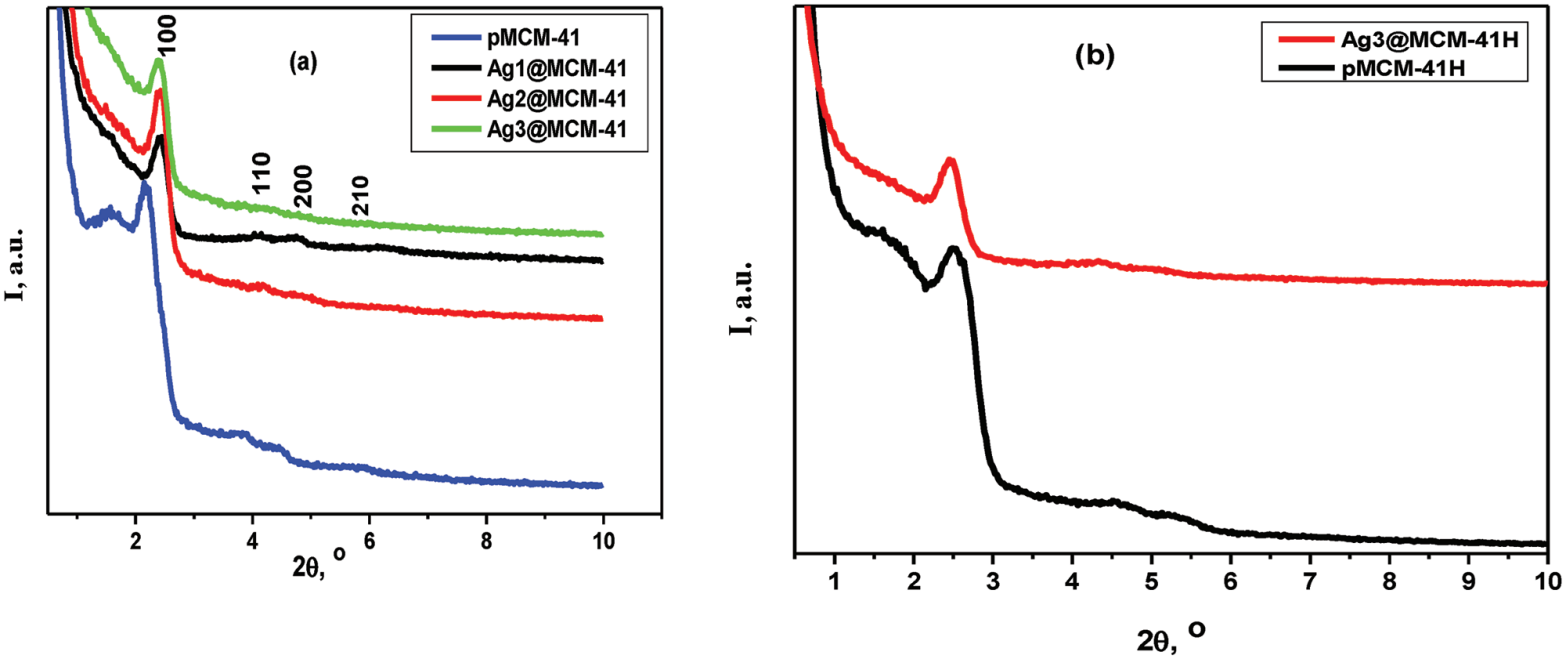
Small angle XRD patterns of a) uncalcined silica composites, b) pMCM‐41H, and Ag3@MCM‐41H.

**Figure** **1**a presents the small‐angle X‐ray diffraction (SAXRD) patterns of the uncalcined silica composites. The pMCM‐41 diffractogram shows four typical peaks of the silica type MCM‐41 corresponding to the reflections of the hexagonal pore arrangement (P6mm space group) of the MCM‐41‐like structure, with *d*_100_ (2θ_1_), *d*_110_ (2θ_2_), *d*_200_, and *d*_210_ at 40.455, 22.765, 19.69 and 14.947 Å, respectively. The diffractograms of the AgNP–silica composites show a slight broadening in the (100) reflection, which is shifted to shorter pore spacings of 36.61, 36.61, and 37.09 Å for the Ag1@MCM‐41, Ag2@MCM‐41, and Ag3@MCM‐41 samples, respectively. The values of sin^2^θ_1_/sin^2^θ_2_ cannot be calculated because the lines of (110), (200), and (210) reflections are almost undetectable, indicating that the AgNP–MCM4‐1 samples have a disoriented unidirectional honeycomb structure. Figure 1b shows the SAXRD diffractograms of the pMCM‐41H and Ag3@MCM‐41H samples, which indicate the maintenance of the same crystal structure but with a slight reduction in *d*_100_ to 35.335 and 35.98 Å, respectively. This reduction may be attributed to the thermal degradation of the surfactant and the partial dehydroxylation of silanol groups, as confirmed by the IR study (Figure S1, Supporting Information). The values of sin^2^θ_1_/sin^2^θ_2_ for pMCM‐41H and Ag3@MCM‐41H are equal to 0.293 and 0.325, respectively, which are close to 1:3, suggesting a long‐range ordered pore arrangement.^48^ The length of the unit cell parameter (*a*_o_) was calculated according to Equation (1), 49

(1) $$a_{o}=2d_{\mathrm{100}}/\sqrt[{2}]{3}$$

The value of the hexagonal cell parameter *a*_o_ is equal to 4.69 nm for pMCM‐41, which is very close to previously reported value.^50^ Upon addition of AgNPs, *a*_o_ dropped to 4.22–4.27 nm. This reduction in *a*_o_ value can be understood in view of the influence of synthesis parameters, such as the hydrogel composition, the surfactant type and chain length, the alkalinity, the temperature, and the synthesis time on the MCM‐41 structure.^51^ After calcination, *a*_o_ decreased to 4.080 and 4.155 nm for pMCM‐41H and Ag3@MCM‐41H, respectively, which can be attributed to the loss of the organic template. These values are within the range of reported values of calcined MCM‐41 (3.8–4.3 nm).^52^ The wide‐angle XRD patterns for the pMCM‐41 and AgNP–MCM‐41 samples before and after calcination are shown in Figure S2 in the Supporting Information. The patterns indicate nearly amorphous silica, as confirmed by the appearance of amorphous humps at 2θ 23°. The AgNP–composites before and after calcination show peaks at 2θ values of 37.96° and 44.44°, corresponding to the (111) and (200) reflections, respectively, whereas the (220) diffraction is hardly observable, probably due to the small size and a possible lesser ordering of AgNPs. However, these may be assigned to the face‐centred‐cubic symmetry of Ag (JCPDS 04‐0873). The appearance of 2θ peaks due to the above‐mentioned planes confirmed the immobilization of AgNPs on silica composites. The diffraction band observed at 2θ 32.25° in some samples may be attributed to remaining traces of sodium chloride (Halite 88‐2300) from the culture salts.

#### 2.1.2 Thermogravimetric Analysis (TGA)

The TGA results for pMCM‐41 and the AgNP‐modified samples are shown in Figure S3 in the Supporting information. The TGA curves exhibit total mass losses for pMCM‐41, Ag1@MCM‐41, Ag2@MCM‐41, and Ag3@MCM‐41 of 42.5%, 40.5%, 40.5%, and 37%, respectively. There are three major mass loss stages in the TGA curves. The first stage shows a mass loss of ≈8% for pMCM‐41 and 4% for uncalcined AgNP–composites below 140 °C, which can be attributed to the loss of adsorbed water.^53^ The second stage is observed between 150 and 300 °C, with a major mass loss of ≈29.4%, 28.7%, 27.2%, and 25.4% for pMCM‐41, Ag1@MCM‐41, Ag2@MCM‐41, and Ag3@MCM‐41, respectively, which is related to the pyrolysis of surfactant (cetyltrimethylammonium bromide, CTAB) cations.^53^ The observed mass loss is in accordance with the observation of the disappearance of IR band near 2900 cm^−1^ which is attributed to CH stretching vibrations (Figure S1, Supporting Information). An elevation of 37–46 °C was observed in the temperature of the surfactant mass loss stage for the modified samples compared with the pMCM‐41. This difference may be explained by the concomitant loss of surface area upon the incorporation of AgNPs into the silica network, which may hinder the loss of the host substrate. The observed decrease in mass loss of the organic content with increasing AgNPs content may be explained by the inclusion of the AgNP micelles during MCM‐41 crystal propagation, which may interrupt the building up of the ordered MCM‐41 structure. Additionally, as the AgNP content increases by increasing the volume of the added AgNPs suspension, the volume/surfactant ratio drifts far from its optimal value, which may diminish micelle and MCM‐41 crystal formation. Consequently, the observed decrease in tunnel width and broadening of XRD reflections for AgNP‐modified samples compared with pMCM‐41 samples may be clarified. The final stage started at 300 °C, with a relatively gradual mass loss, and this loss may be due to the condensation of the silanol groups.

#### 2.1.3 N_2_ Physisorption Studies

**Figure 2:**
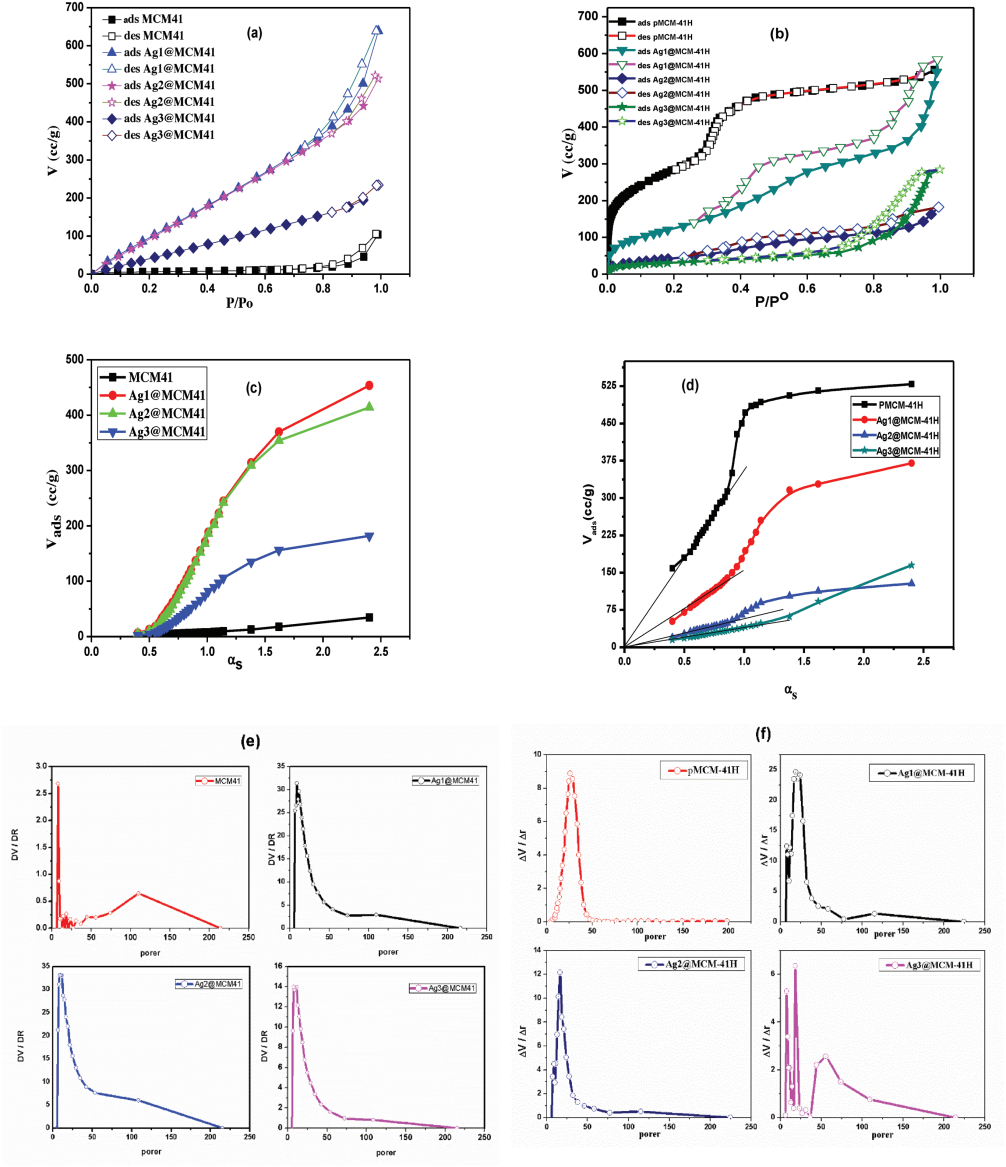
The nitrogen adsorption/desorption isotherms for a) uncalcined samples and b) calcined samples, α_s_‐plots of nitrogen adsorption for c) uncalcined samples and d) calcined samples and pore volume distribution of e) uncalcined samples and f) calcined samples.

To evaluate the textural properties of the samples, a N_2_ physisorption analysis was performed. **Table** **1** summarizes the textural properties of the pMCM‐41 and AgNP–composites before and after calcination. For the uncalcined composites, the N_2_ adsorption–desorption isotherms are shown in **Figure** **2**a. The isotherms show that the curves are attributable to type II isotherm and H3 hysteresis loops.

**Table 1:** Composition and textural properties of the studied samples

Sample	Ag [%]	*S*_BET_ a) [m^2^ g^−1^]	*V*_0.95_ b) [cc g^−1^]	*V*_T_ c) [mL g^−1^]	*ȓ* d) [Å]	*C* e)	*S*_α_ f) [m^2^ g^−1^]	*S*_meso_ g) [m^2^ g^−1^]	*S*_non‐meso_ h) [m^2^ g^−1^]
pMCM‐41	–	21.32	61.11	0.0945	88.7	111.33	21.7	–	21.7
Ag1@MCM‐41	0.034	611.12	536.79	0.83	27.17	4.76	600	40	560
Ag2@MCM‐41	0.251	693.65	457.02	0.707	20.38	3.44	650	134	516
Ag3@MCM‐41	0.369	304.1	203.96	0.3155	20.75	3.45	300	30	270
pMCM‐41H	–	1020	541.25	0.86	16.828	151.29	1026	1019	7
Ag1@MCM‐41H	0.053i)	482.14	423.21	0.6547	27.00	40.51	480	470	10
Ag2@MCM‐41H	0.384i)	174.80	146.82	0.227	26.00	30.3	170	157	13
Ag3@MCM‐41H	0.543i)	111.96	238.94	0.696	124.3	100.51	111	40	71

^a)^ *S*_BET_: total surface area according to Brunauer, Emmett, and Teller

^b)^ *V*_0.95_: The total pore volume

^c)^ *V*_T_: the volume of liquid nitrogen

^d)^ *r*^: mean pore diameter

^e)^ *C*: constant related to the heat of adsorption

^f)^ *S*_α_: the specific surface area related to α_s_ method

^g)^ *S*_meso_: the mesopore surface area related to α_s_ method

^h)^ *S*_nonmeso_: the nonporous surface area related to α_s_ method

^i)^ Calculated according to TGA residues at 400 °C and Ag% for uncalcined samples.

The α_s_ curves and pore size distribution of the uncalcined composites are shown in Figure 2c,e, respectively. The results of these measurements show that the pMCM‐41 sample has a very small surface area (*S*_BET_ = *S*_nonmeso_ ≈ 21.3 m^2^ g^−1^), which is related to the nonmesoporous structure. The highly ordered hexagonal pore structure of MCM‐41 cannot yet be observed as its pores are occupied with CTAB. Moreover, the values of *r*ˆ = 88.7 Å and *V*_T_ = 0.094 mL g^−1^ for pMCM‐41 indicate the formation of a macroporous structure. However, the ordered honeycomb structure of MCM‐41 was adequately verified by SAXRD study.

Upon the immobilization of AgNPs, the surface area of the AgNP–silica composites substantially increased compared with the pMCM‐41, which is probably attributable to the formation of a microporous structure.

In the case of the calcined samples, pMCM‐41H shows a large surface area (*S*_BET_ = 1020 m^2^ g^−1^) and a large total pore volume (*V*_T_ = 0.86 mL g^−1^), which are comparable to the values reported in the literature.^49^, ^54^ The large surface area is due to the relatively large internal surface area (*S*_meso_ = 1019 m^2^ g^−1^) of the honeycomb‐mesoporous structure. The addition of AgNPs during the preparation causes remarkable structural perturbations. The total surface area decreases to 482.14, 174.8, and 111.96 for Ag1@MCM‐41H, Ag2@MCM‐41H, and Ag3@MCM‐41H, respectively. The mean pore diameter (*r*ˆ) increases from 16.82 to 26.0–124.3 nm upon the incorporation of AgNPs. This increment may be attributed to the plugging of smaller pores by AgNPs, as well as the formation of less‐ordered honeycomb‐like mesopores, which also leads to a decline in the surface area. It is noteworthy that the large effect of AgNPs on the textural properties of composites is due to the applied suboptimal volume/surfactant ratio. The total pore volume (*V*_T_) of the calcined samples varies from 0.86 to 0.69 mL g^−1^, indicating the differences in the pore volume and the mesopore shape.

The N_2_ adsorption/desorption isotherms of the calcined samples in Figure 2b show a typical type IV isotherm and H1 hysteresis loop, based on the empirical IUPAC classification, which are indicative of mesoporous materials.^55^ The point of inflection at a relative pressure of *P*/*P* = 0–0.3 represents the completion of monolayer coverage by the N_2_ adsorbate. The uptake in N_2_ adsorption from *P*/*P* = 0.3–0.4 is corresponding to the N_2_ condensation within the ordered mesopores of pMCM‐41H. The steepness of this step (*P*/*P* = 0.3–0.4) emphasizes the narrow ordered pore size distribution of typical mesoporous materials. It is worth noting that the steepness decreases and moves to higher *P*/*P* values upon the incorporation of AgNPs, confirming some loss of the ordered structure upon metal addition. Additionally, the hysteresis loop widens with increasing Ag content, which indicates that AgNPs disturb the building up of typical MCM‐41 cylindrical pores. The plateau at high relative pressures (*P*/*P* = 0.4–0.85) is attributable to the multilayer N_2_ adsorption. Finally, the sharp N_2_ adsorption at higher relative pressures (*P*/*P* > 0.9) associated with a small hysteresis loop is usually ascribed to N_2_ condensation in the interparticle pores.^56^

Figure 2d shows the α_s_ plots of the synthesized samples after calcination. For α_s_ > 1, the slope of the straight line which does not pass through the origin but intersects the *V*_α_ axis is used to calculate the mesoporous surface area (*S*_meso_), while the slope of the straight line passing through the origin is used to determine the total surface area (*S*_α_). The surface area values (*S*_nonmeso_, *S*_meso_, and *S*_α_) are given in Table 1. *S*_α_ is always comparable to *S*_BET_. Figure 2f shows the pore size distributions determined according to the Barrett, Joyner, and Halenda (BJH) method for the calcined composites. The narrow distribution for pMCM‐41H sample indicates its uniform mesoporous structure. The calcined AgNP‐doped samples are characterized by a small shift toward the microporous structure that increases from Ag1@MCM‐41H to Ag2@MCM‐41H. The further increase in Ag content in Ag3@MCM‐41H shows a greater shift toward the microporous structure and the pore widening.

It seems that pMCM‐41H has a very high surface area and that most of the existing pores are mesoporous; however, with increasing amounts of Ag, some pores become nonmesoporous, as shown by increasing values of *S*_nonmeso_. Thus, it can be concluded that the presence of a large number of AgNPs distributed along the honeycomb‐like cylinders or blocking their entrances influences the pore volume. The values of *r*ˆ = 88.7 Å and *V*_T_ = 0.094 mL g^−1^ for pMCM‐41 indicate the nonmesoporous structure, while the values of *r*ˆ = 16.82 Å and *V*_T_ = 0.86 mL g^−1^ for pMCM‐41H indicate the mesoporous structure. On the other hand, comparing the textural properties of the calcined and uncalcined samples, which have the same levels of AgNP addition, reveals that the *S*_BET_ values of the uncalcined samples are greater than those of the calcined samples, while the *S*_meso_ values of the uncalcined samples are less than those of the calcined samples.

The main pore diameter (*r*ˆ_0.5–5.0_) in the range from 0.5 to 5 nm was calculated (**Table** **2**) from the pore distribution curves (Figure 2e,f); *r*ˆ_0.5–5.0_ increases with the immobilization of AgNPs in Ag1@MCM‐41 compared to pMCM‐41. Further increment in AgNP immobilization decreases *r*ˆ_0.5–5.0_, as observed for Ag2@MCM‐41 and Ag3@MCM‐41. Regarding the actual size of AgNPs (7–48 nm), the observed *r*ˆ_0.5–5.0_ values (1.089–0.972 nm), AgNPs cannot be hosted in the tubular MCM‐41 structure, and this may explain the observed dislocation of the honeycomb arrangement. The MCM‐41H and Ag3@MCM‐41H samples showed larger pores than did their parent materials due to the loss of the organic template.

**Table 2:** Physical properties of pure and AgNP–composites

Sample	*d*_100_ [nm]	*a*_o_ [nm]	*ȓ*_0.5–5.0_ a) [nm]	*W*_t_ [nm]
pMCM‐41	4.061	4.690	0.791	3.899
Ag1@MCM‐41	3.655	4.220	1.089	3.131
Ag2@MCM‐41	3.652	4.216	0.999	3.217
Ag3@MCM‐41	3.696	4.268	0.972	3.296
pMCM‐41H	3.534	4.080	2.709	1.371
Ag3@MCM‐41H	3.598	4.155	1.832	2.323
			0.766	3.389

^a)^ Meso‐ or micropore diameter.^52^

By combining the XRD and surface area data, the wall thickness (*W*_t_) of the honeycomb‐like structure can be calculated according to formula (Equation (2)), as detailed in Table 2.^57^

(2) $$W_{t}=a_{o}\;-D_{p}$$

*W*_t_ of pMCM‐41 is threefold that of pMCM‐41H due to the silanol condensation and the healing of micropores, which is typical for the MCM‐41 structure. Although the uncalcined AgNP–composites showed a smaller *W*_t_, which with increasing AgNP content, the calcined Ag3@MCM‐41 exhibited two thicker types of walls similar to its uncalcined form. This may be understood regarding the relative decrease in the surface area noted with increasing AgNP content in silica composites (Table 1) and the decrease in the loaded organic template. According to these studies, AgNPs are suggested to be caged in the mesopores of the disordered confinements within silica composites, as illustrated in the scheme shown in Figure S4 in the Supporting Information.

#### 2.1.4 Scanning Electron Microscopy (SEM)

**Figure 3:**
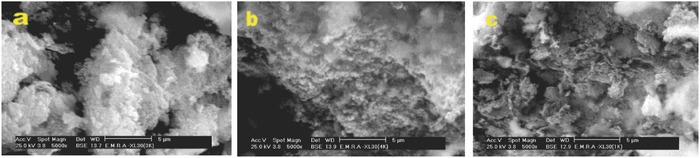
SEM images of a) MCM‐41, b) Ag3@MCM‐41, and c) Ag3@MCM‐41H.

The SEM images of pMCM‐41, Ag3@MCM‐41, and Ag3@MCM‐41H are presented in **Figure** **3** at ×5000 magnification. All samples are aggregates of silica sheets with a porous structure. The SEM image of pMCM‐41 shows a large particle size with a relative smooth surface, which leads to small surface area compared to Ag3@MCM‐41 which is characterized with the same particle size but a rougher surface. This roughness may account for the great increase in the surface area. The Ag3@MCM‐41H sample shows aggregates of larger particles than those of the corresponding uncalcined samples, in addition to a rough surface resulting from calcination, indicating that the immobilization of AgNPs on MCM‐41 and the calcination temperature influence the surface morphology. The SEM image observations are in agreement with the results of the textural analysis.

#### 2.1.5 Transmission Electron Microscopy (TEM)

**Figure 4:**
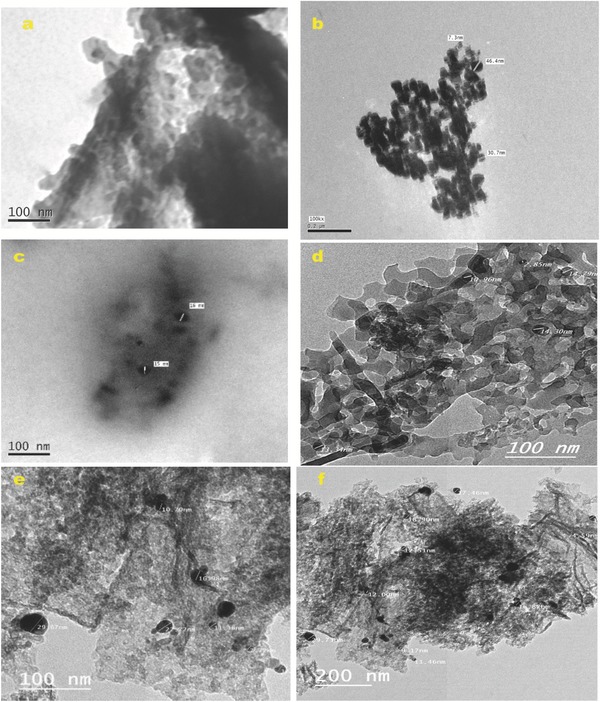
TEM images of a) pMCM‐41, b) AgNPs (scale bar 0.2 µm), c) Ag1@MCM‐41, and d) Ag3@MCM‐41 e,f) Ag3@MCM‐41 after 58 months at different scale.

TEM images of the AgNP‐composite suspensions are shown in **Figure** **4**. The TEM image of the pMCM‐41 sample (Figure 4a) clearly shows tubular arrays of MCM‐41. TEM investigation of the AgNPs (Figure 4b) shows the accumulation of AgNPs in the form of dark aggregates with a size range of ≈7–48 nm. TEM images of Ag1@MCM‐41 and Ag3@MCM‐41 (Figure 4c,d, respectively) show two silica structures; i) the tubular honey comb structure of MCM‐41 and ii) a disordered amorphous silica phase sequestrating dark, spherical, and elongated spots corresponding to AgNPs ranging in size from 10 to 20 nm. No agglomeration of AgNPs is shown, indicating that the embedment of AgNPs on silica composites prevents the aggregation and improves the colloidal stability of the AgNPs.

To investigate the long‐term stability of AgNPs in the AgNP–MCM‐41 samples, changes in the size distribution of the AgNPs were analyzed using TEM after ≈5 years of storage, and the results are given in Figure 4e,f. Surprisingly, the average AgNP size of the Ag3@MCM‐41 composite (stored under ambient conditions) remained approximately constant. Furthermore, AgNPs remained sequestrated in the disordered confinement of the silica/MCM‐41 composites without agglomeration. An important finding from these studies is that this strategy for the immobilization of AgNPs using MCM‐41 as inorganic support provides high dispersity and stability of AgNPs and prevents their agglomeration for long period.

### 2.2 Antimicrobial Activity

#### 2.2.1 Cup–Plate Method

The antimicrobial activity of the AgNP–composites before and after calcination compared to pMCM‐41 was individually evaluated against *Staphylococcus aureus*, *Escherichia coli*, and *Candida albicans* using the cup–plate method based on the zone of inhibition. As expected, the pMCM‐41 matrix did not show any antimicrobial activity toward the tested microbes. The uncalcined AgNP–composites exhibit excellent antimicrobial activity against all the tested microbes. The diameters of the inhibition zones are shown in **Table** **3**. The inhibition zone diameters increased with increasing AgNPs load within the uncalcined silica composites and with increasing specific surface area. Increasing AgNP content enhances the driving force of Ag^+^ diffusion from the bulk to the surface, which is favored due to the versatile hydrophilic porous silica surface structure and possible diffusion assistance from the intercalated CTAB. The antimicrobial functionality of the AgNP‐modified silica samples is better than that of many reported modified materials, as detailed in Table S1 in the Supporting Information. Repeating these investigations for the Ag2@MCM‐41 and Ag3@MCM‐41 samples, after storage for ≈5 years under ambient conditions, yielded almost the same antimicrobial activity, indicating the superior sustainability of AgNP–composites.

**Table 3:** Antimicrobial activity of uncalcined samples

Samples	Inhibition zone [mm]		
	*Staphylococcus aureus*	*Escherichia coli*	*Candida albicans*
pMCM‐41	–	–	–
Ag1@MCM‐41	12	12	11
Ag2@MCM‐41	13	15	12
Ag3@MCM‐41	18	19	17
Ag2@MCM‐41a)	14	15	14
Ag3@MCM‐41a)	18	18	20

^a)^ After 58 months of storing.

The calcined samples showed no antimicrobial activity. These results could be expected because the caging of AgNPs inside the disordered amorphous silica limits their release, and the diffusion of silica through the culture is hampered by the effective silanol condensation during calcination and the loss of diffusion assistance from the surfactant, which is thermally degraded. Thus, AgNPs should be free to diffuse from the silica host to exert antimicrobial activity, as is the case for uncalcined AgNP–composites.

#### 2.2.2 Plate‐Count Technique

**Figure 5:**
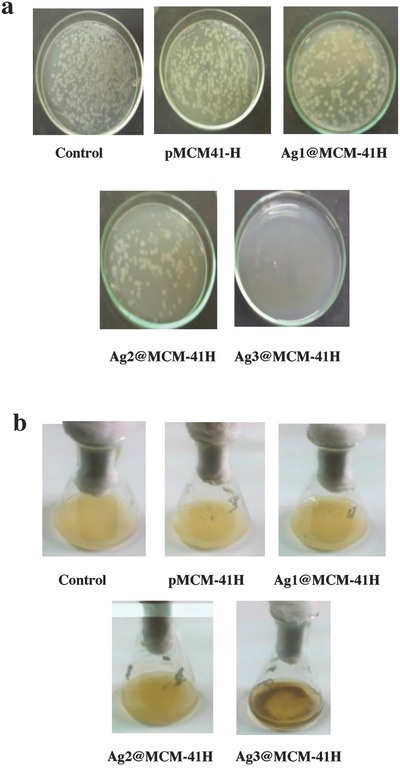
a) *Staphylococcus aureus* colony forming units of control, culture medium, pMCM‐41H, and calcined AgNP–composites, b) their growth density of *Staphylococcus aureus* cultures.

The bactericidal effects of the calcined samples against *S. aureus*, one of the most virulent strains found in the clinic in biomaterial‐associated infections, were investigated using the plate‐count technique instead of the static cup–plate method due to the observed diffusion inactivity of the calcined samples. The results are shown in **Table** **4** and **Figure** **5**a,b and indicate the following: a) the pMCM‐41H sample exhibited no antibacterial activity; b) the rate of bacterial reduction increased with increasing AgNP content; c) the Ag1@MCM‐41H and Ag2@MCM‐41H samples showed significant antibacterial activity, with reduction rates of 51.56% and 58.48%, respectively; d) *S. aureus* growth is completely inhibited by Ag3@MCM‐41H, reflecting the key role played by the greater Ag content within silica composites in the antibacterial activity; and e) the colony‐forming unit (CFU) results of the studied samples are consistent with the optical density (OD) measurements, which show a lower OD for Ag3@MCM‐41H than for Ag1@MCM‐41H and Ag2@MCM‐41H.

**Table 4:** Antibacterial activity of calcined samples in comparison to control (*Staphylococcus aureus* culture)

Sample	Absorbance at 660 nm	Number of CFU [10^7^ mL^−1^]	Reduction in CFU [%]
Bacterial culture	1.638	2.89	0.0
pMCM‐41H	2.002	2.78	3.80
Ag1@MCM‐41H	1.521	1.4	51.56
Ag2@MCM‐41H	1.335	1.2	58.48
Ag3@MCM‐41H	0.450	0	100.00

## 3 Conclusions

In the present study, a green and a facile approach is presented to immobilize AgNPs on MCM‐41. This strategy can prevent AgNP aggregation and enable continuous AgNP release from MCM‐41‐based composites. The successful AgNP immobilization is demonstrated by flame atomic absorption spectrometry (FAAS), SAXRD, Fourier‐transform infrared spectroscopy (FTIR), XRD, TGA, SEM, and TEM. The SAXRD study shows that the intensity of the (100) reflection in the Ag‐doped samples decreases with increasing Ag%, indicating that the tunnel population decreases and that AgNPs are hosted within the disordered amorphous silica rather than in nanotunnels. In contrast, the average pore size increases from 16.82 to 124.3 nm upon incorporation of the AgNPs, which can be attributed to the establishment of AgNP‐centered disordered silica at the expense of the ordered honeycomb‐like structure. The synthesized samples were also examined for their antimicrobial activity. The results of the cup–plate method show that the Ag3@MCM‐41 composite exerts the best antimicrobial activity against the entire range of the tested microbes due to the higher content of AgNPs compared with the Ag1@MCM‐41 and Ag2@MCM‐41 composites.

Thermal treatment of the AgNP‐modified samples diminishes AgNP release from the MCM‐41 such that they exert no antimicrobial activity via the cup–plate method, which is mainly attributable to diffusion issues. Comparison of the antibacterial activity of calcined samples using CFU method clearly indicated that Ag3@MCM‐41H composite showed the best antibacterial activity with the bacteria mortality reaching 100% compared with Ag1@MCM‐41H and Ag2@MCM‐41H. The results of this study reflect that calcined and uncalcined Ag3@MCM‐41 composites are potential candidate materials which offer an ideal AgNPs content, optimum silica surface area, pore versatility, and stability. Therefore, these materials can be utilized effectively as an economic material in different antimicrobial applications, such as sustainable disinfection reagents or additives for potential wound dressing.

## 4 Experimental Section

*Materials*: Unless otherwise specified, all chemicals were of analytical reagent grade and used as received. Double‐ distilled water was used throughout the experiments. CTAB was purchased from Sigma‐Aldrich (USA). Sodium trisilicate (NaSi_3_O_7_) (10% H_2_O) was obtained from Fluka, Switzerland. Silver nitrate (AgNO_3_) was supplied from Merck, Germany. *Macrococcus bovicus* was locally isolated from the soil and employed in the biosynthesis of AgNPs from AgNO_3_ with a size range of ≈7–48 nm, as previously described.^35^

*Instrumentation*: The infrared spectra of samples were measured by FTIR on a Perkin Elmer spectrophotometer in the range of 4000–400 cm^−1^ using KBr discs. Surface area measurements were performed on a Quantachrome USA NOVA 22 system by gaseous N_2_ adsorption at −196 °C. The samples (0.1–0.2 g) were degassed at 300 °C for 3 h. A relative pressure range of 0.05–0.25 was used in the surface area analysis. The α_s_ method was used as a simple and effective way to analyze the surface area;^58^ additionally, the modeless pore size analysis method was used to determine the pore volume distribution of the synthesized samples.^54^, ^59^ Computations were carried out starting from *P*/*P* = 0.95 down to *P*/*P* = 0.3 to cover the hysteresis region of adsorption completely. TGA of dry powdered samples was performed on an SDTQ600 (TA Instruments, USA) thermal analyzer at a heating rate of 10 °C min^−1^ between room temperature and 700 °C under a nitrogen atmosphere. Powder XRD patterns of the studied samples were obtained by a Bruker AXS D8 ADVANCE using Cu Kα radiation (λ = 1.5406 Å) under 40 kV and 30 mA. The SEM images were obtained by an FEI Quanta‐250 SEM (FEI Company, The Netherlands) equipped with an energy dispersive X‐ray analysis unit operating at 30 kV. The TEM measurements were carried out using a JEOL model 1200EX electron microscope at an operating voltage of 120 kV. TEM measurements were performed by drop‐coating the synthetized samples onto carbon‐coated copper grids (40 µm × 40 µm mesh size). The samples were then dried and kept under vacuum prior to being loaded into a specimen holder. FAAS (Analyst 800, PerkinElmer USA) was employed for the determination of Ag concentration in the synthesized samples. The samples were digested in HF/HNO_3_ in a Milestone MLS Ethos 900 Plus Microwave prior to this analysis.

*Synthetic Procedures—Immobilization of AgNPs on MCM‐41*: The MCM‐41 was synthesized according to the procedure reported in the literature, with some modifications.^53^ Sodium trisilicate was employed as a source of silicon, and CTAB was used as a surfactant template. Typically, 2.4 g of CTAB was dissolved in 46 mL of double‐distilled water and stirred for 15 min. Then, 4.568 g of NaSi_3_O_7_ was dissolved in 46 mL of 0.3 mol L^−1^ NaOH solution and added to the CTAB solution dropwise; the mixture was vigorously stirred for 30 min. To prepare various AgNP–MCM‐41 samples, a series of 1.5 m mol L^−1^ AgNP suspensions with volumes 60, 120, and 240 mL were separately added to the mixtures, and the stirring was continued for ≈30 min. The pH was adjusted to 9.2 ± 0.1 using 2 mol L^−1^ H_2_SO_4_, and the vigorous stirring was continued for an additional 24 h. Bulky, pale reddish‐brown gels formed. The resulting gels were allowed to stand for 24 h at room temperature. Finally, the samples were centrifuged, washed three times with double‐distilled water, and dried at 50 °C. According to the volume added from the AgNP suspensions (60, 120, and 240 mL), the samples were coded as pMCM‐41, Ag1@MCM‐41, Ag2@MCM‐41, and Ag3@MCM‐41, respectively. For further studies, these samples were also calcined at 400 °C for 6 h in air and named pMCM‐41H, Ag1@MCM‐41H, Ag2@MCM‐41H, and Ag3@MCM‐41H. The synthesized composites without AgNPs before and after calcination were named as pMCM‐41 and pMCM‐41H, respectively. The samples were stored at ambient conditions of light and temperature in closed transparent and colorless glass bottles which were repeatedly subjected to air during the withdrawing of samples for analysis.

*Synthetic Procedures—Antimicrobial Activity of AgNP‐modified MCM‐41*: The pMCM‐41 and pMCM‐41H samples were used as controls for the antibacterial activity experiments. The cup–plate method was utilized to evaluate the antimicrobial activity of the studied samples against strains of bacteria (Gram positive, *Pseudomonas aeruginosa*; Gram negative, *E. coli*) and yeast (*C. albicans*). The test microbes were inoculated into 20 mL of sterile nutrient broth and incubated at 37 °C for 16–18 h. Using a sterile cotton swab, the nutrient broth cultures were swabbed on the surface of sterile nutrient agar plates. Agar wells were prepared with the aid of a sterilized cork borer with 10 mm in diameter.^60^ Then, 0.1 g of the samples and the controls was separately suspended in 1 mL of double‐distilled water, and 100 µL of these suspensions was added to the different wells in the plate. Subsequently, these plates were incubated in an upright position at 37 °C for 24 h. Finally, the antimicrobial activity was estimated by measuring the diameter of the zone of inhibition in mm against the tested organism.

Furthermore, the antimicrobial activity of the calcined samples was examined using plate count technique.^35^ The inocula of *S. aureus* was prepared by growing the strains in a freshly prepared liquid nutrient broth which containing 5 g L^−1^ peptone and 3 g L^−1^ beef extract at pH 6.8 and incubated for 24 h. In the antibacterial activity test, 0.05, 0.1, or 0.25 g of the sample or the control were added in separate batches to the inoculated flasks. Then, the flasks were incubated at 37 °C for 16 h. Serial dilution from sample‐containing culture and the control has been done (10^−2^–10^−7^). The microbial inhibition was evaluated by two approaches. First, 100 µL from each dilution has been spread over the surface of a nutrient medium solidified with agar in a petri dish and the number of CFU was counted. The reduction growth rate (*R*) for treated samples in relation to control was calculated using Equation (3)

(3) $$R\;\left(\%\right)\;=\;\frac{B-A}{B}\;\times \;100$$

where *A* is CFU mL^−1^ for the treated sample after 16 h of incubation, and *B* is CFU mL^−1^ for the control sample after 16 h of incubation.^61^ In the second approach, the growth rates were determined by measuring the OD at 660 nm of the incubation liquid culture medium. The greater the growth, the higher the turbidity; the OD figure was directly proportional to the number of bacteria in the medium.

## Conflict of Interest

The authors declare no conflict of interest.

## Supporting information

Supplementary
