# Supplementary material for: Encapsulation of Biosynthesized Nanosilver in Silica Composites for Sustainable Antimicrobial Functionality
Source: Glob Chall. 2018 Aug 19;2(10):1800048. doi: 10.1002/gch2.201800048 (PMC6607213; doi:10.1002/gch2.201800048)

Copyright WILEY-VCH Verlag GmbH & Co. KGaA, 69469 Weinheim, Germany, 2018.

Global Challenges
Open Access

Supporting Information

for *Global Challenges,* DOI: 10.1002/gch2.201800048

Encapsulation of Biosynthesized Nanosilver in Silica Composites for Sustainable Antimicrobial Functionality

Khaled S. Abou-El-Sherbini, Mohey H. A. Amer, Mohamed S. Abdel-Aziz, Esmat M. A. Hamzawy, Walid Sharmoukh, and Mohamed M. Elnagar*

Supporting Information for

Encapsulation of Biosynthesized Nano-Silver in Silica Composites for Sustainable Antimicrobial Functionality

Khaled S. Abou-El-Sherbini[a*], *Mohey H. A. Amer*[b], *Mohamed S. Abdel-Aziz*[c], *Esmat M. A. Hamzawy*[d], *Walid Sharmoukh*[a], *Mohamed M. Elnagar*[a]

[a]Department of Inorganic Chemistry National Research Centre, 33 El Bohouth St. (former Tahrir St.), 12622, Dokki, Giza, Egypt

[b]Higher institute of Engineering and Technology, kilo 112 Cairo Alex Agricultural Road, Tanta, Egypt

[c]Department of Microbial Chemistry National Research Centre, 33 El Bohouth St. (former Tahrir St.), 12622, Dokki, Giza, Egypt

[d]Department of Glass, National Research Centre, 33 El Bohouth St. (former Tahrir St.), 12622, Dokki, Giza, Egypt

[*]Corresponding author: email: kh_sherbini@yahoo.com

FT-IR investigations

The FT-IR spectra of pMCM-41 and AgNP–composites before and after calcination in the range of 4000–400 cm^{-1} are depicted in Fig. S1 (a, and b, respectively). In the FT-IR spectra of both calcined and uncalcined samples, bands are observed at about 1654 cm^{-1} (O–H bending), 1000-1250 cm^{-1} (Si–O–Si stretching, broad), 962 cm^{-1} (Si–OH stretching), 790 cm^{-1} (Si–O–Si bending) and 451 cm^{-1} (Si–O–Si rocking). Also, the broad and strong band near 3433 cm^{-1} is ascribed to bridged hydroxyl groups and trapped water molecules. [S1] Furthermore, the uncalcined samples exhibit absorption bands around 2922, 2852, 1481 cm^{-1} which may be assigned to asymmetric and symmetric C–H stretching and C–H bending vibrations of the surfactant molecules. [S2] The intensity of these bands was observed to slightly decrease with increasing AgNPs content indicating a slight parallel decrease in the surfactant content. The disappearance of these bands in the calcined samples indicates that the calcination removes most of the template used in synthesis of pMCM-41. The appearance of weak band at 1460 - 1420 cm^{-1} after calcination is due to the CTAB residue. Otherwise, the band attributed to Si–OH stretching and is observed to be weakened and broadened indicating a loss in silanol groups due to thermal dehydroxylation. The spectra of AgNP–composites do not reveal new peaks different from the case of unmodified sample which indicated that the fundamental structure of MCM41 was not changed. This may clarify that the immobilization of AgNPs on silica composites mainly results from physical embedment process. However, an interaction between AgNPs with the surfactant molecules cannot be totally excluded in view of the observation of an enhancement of the IR absorption band at 1383 cm^{-1} assigned to -C-H stretching vibration [S3] and the splitting of the band at 1654 cm^{-1} as well as the red shift of the bands 1480-1490 cm^{-1} with the increase of AgNPs percentage.

a-

pMCM-41

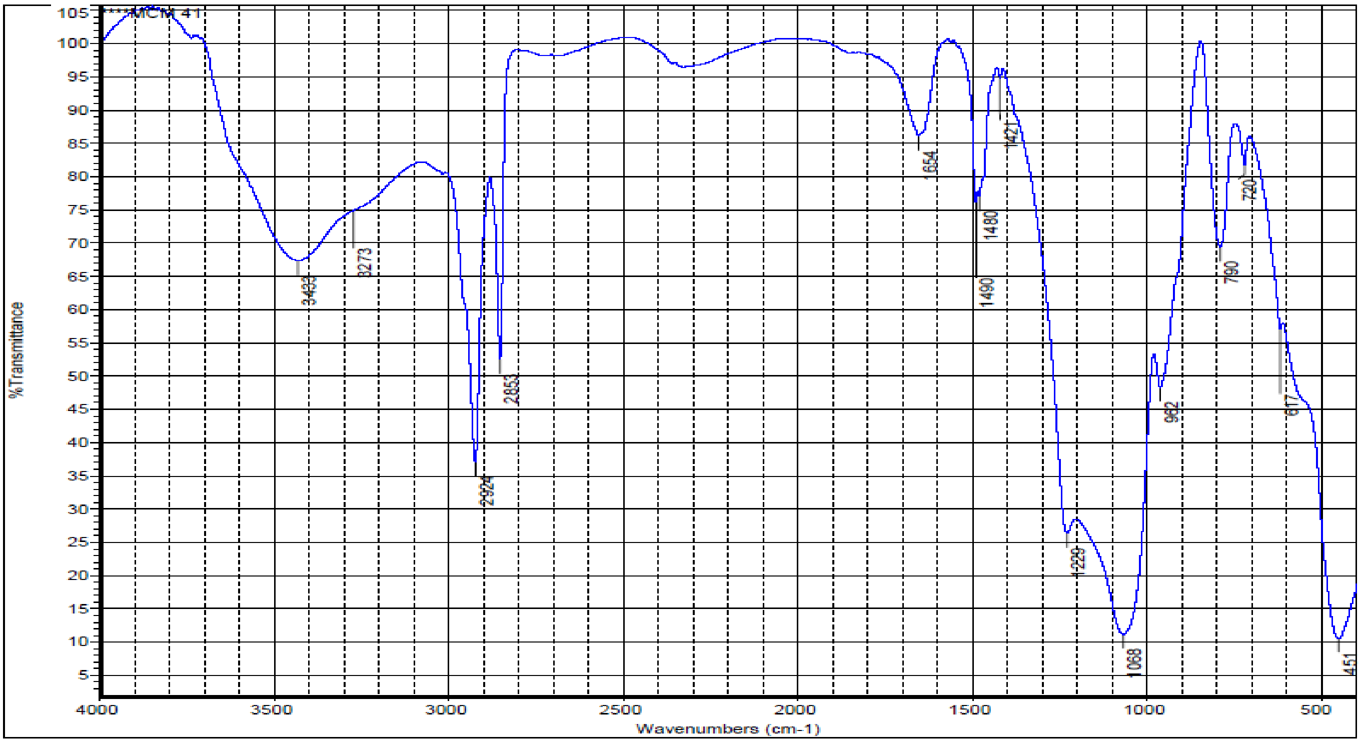

Ag1@MCM-41

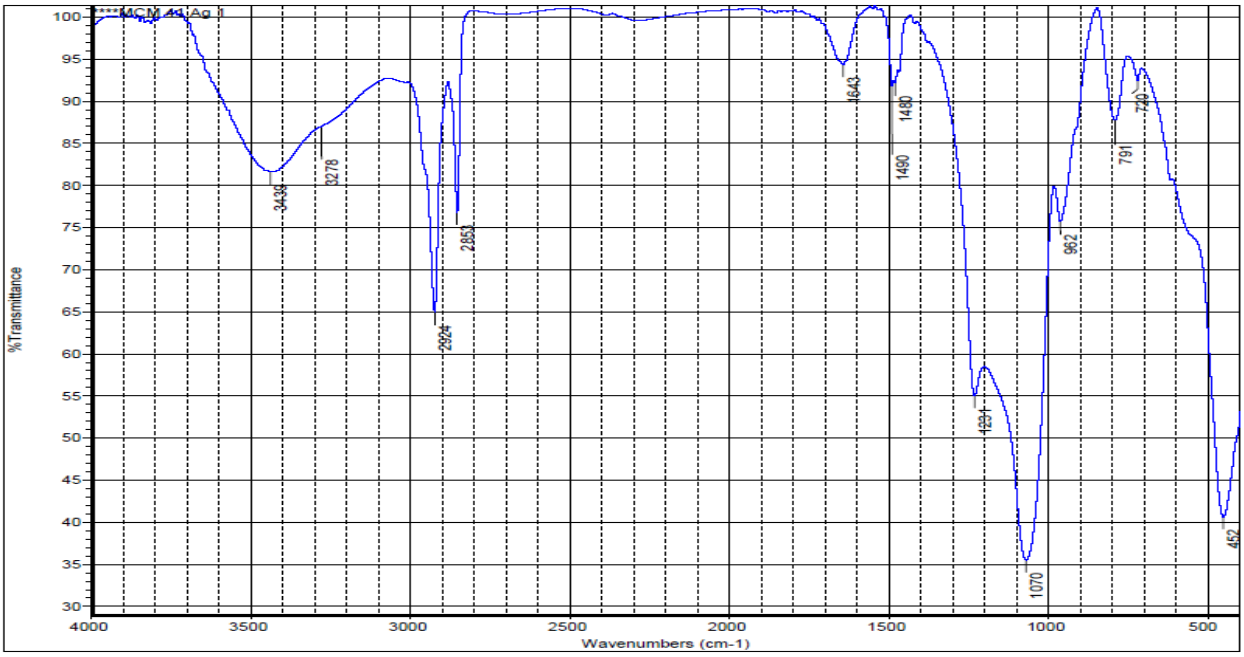

Ag2@MCM-41

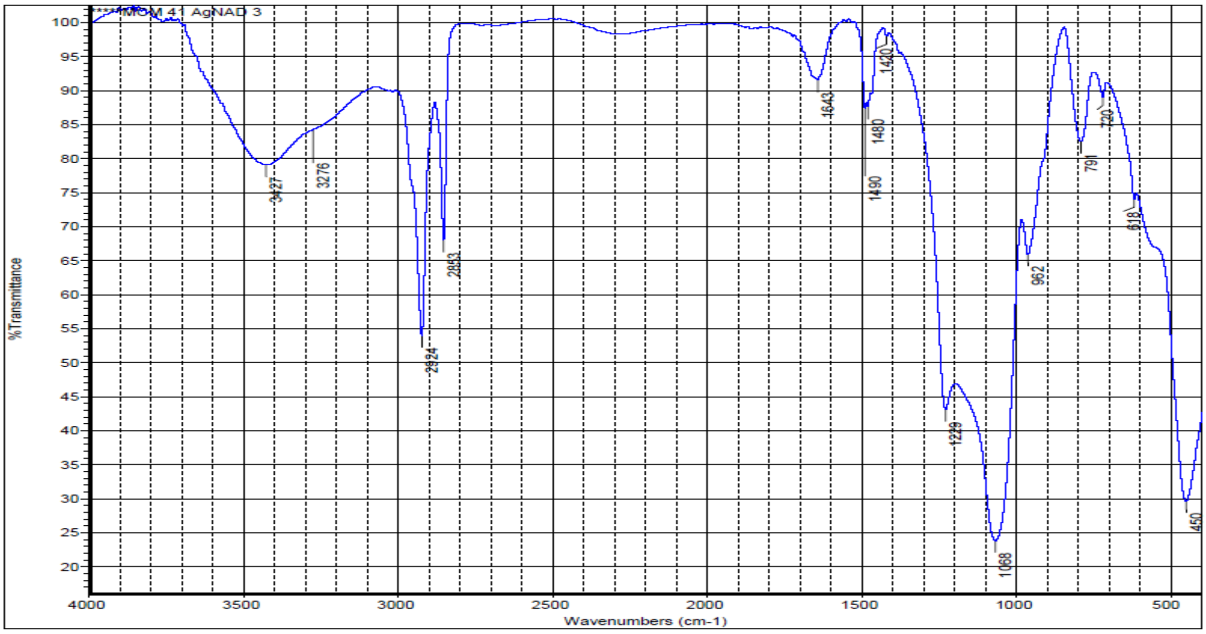

Ag3@MCM-41

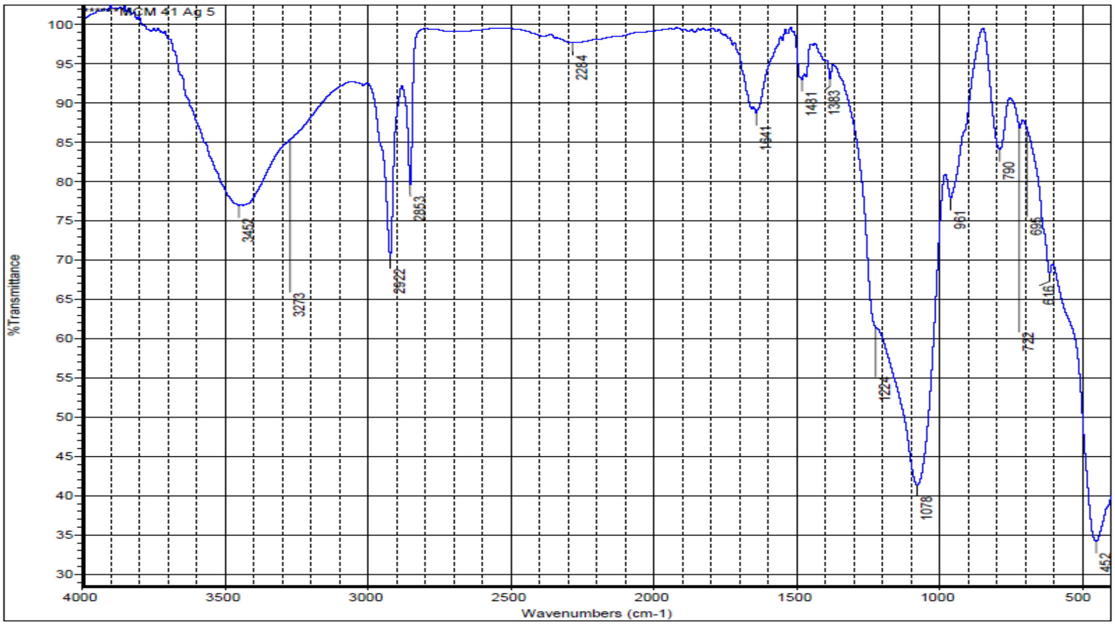

5

b-

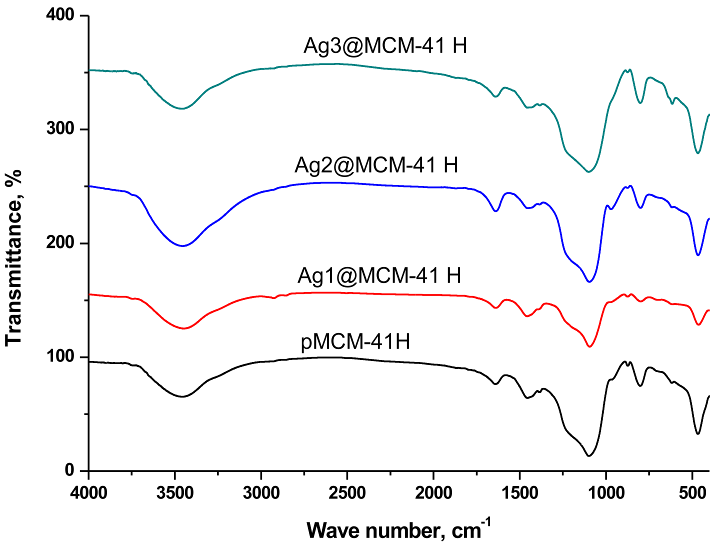

Fig. S1. FT-IR spectra of (a) uncalcined (b) calcined pMCM41 and AgNP- silica composites.

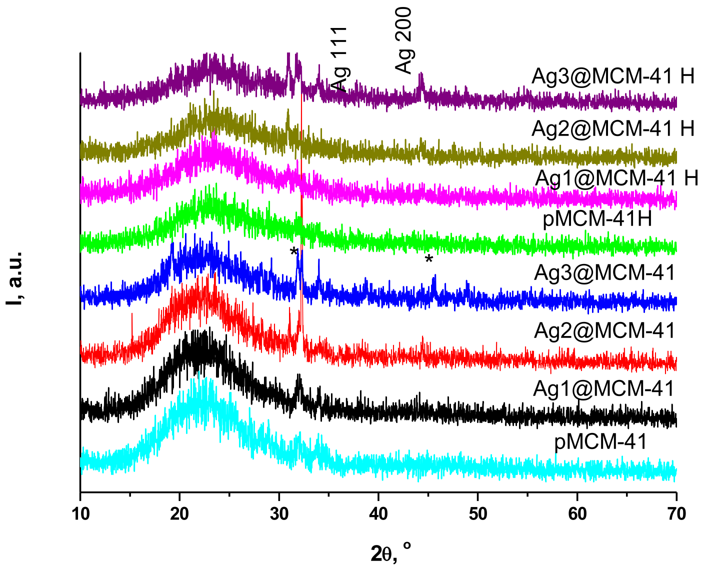

Fig.S2. XRD patterns of AgNP– silica composites

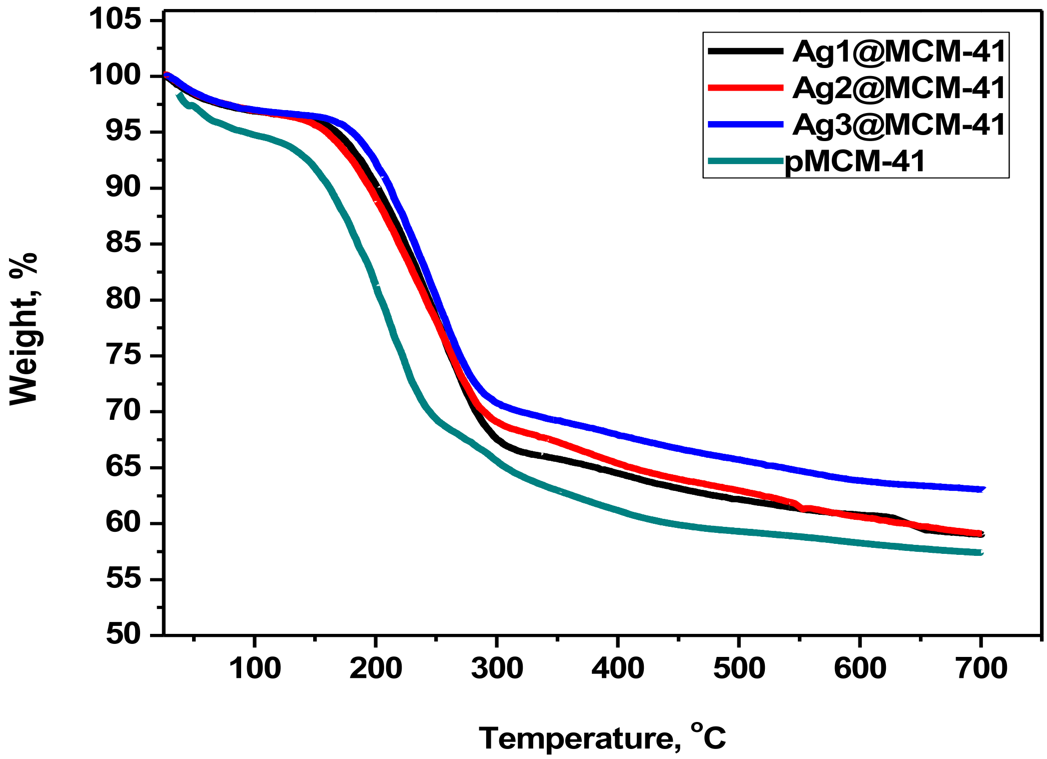

Figure S3. Thermogravimetric analysis of pMCM-41 and AgNP–silica composites

Flame AAS measurements

Flame AAS analysis of the AgNP–composites confirmed Ag embedment at concentrations of 0.034, 0.251 and 0.369% in Ag1@MCM-41, Ag2@MCM-41 and Ag3@MCM-41, respectively. These results indicate that the lowest immobilization efficiency was obtained for those samples immobilized with 60 mL of AgNP suspension. However, the highest immobilization efficiency was obtained for those samples modified with 240 mL of AgNP suspension.

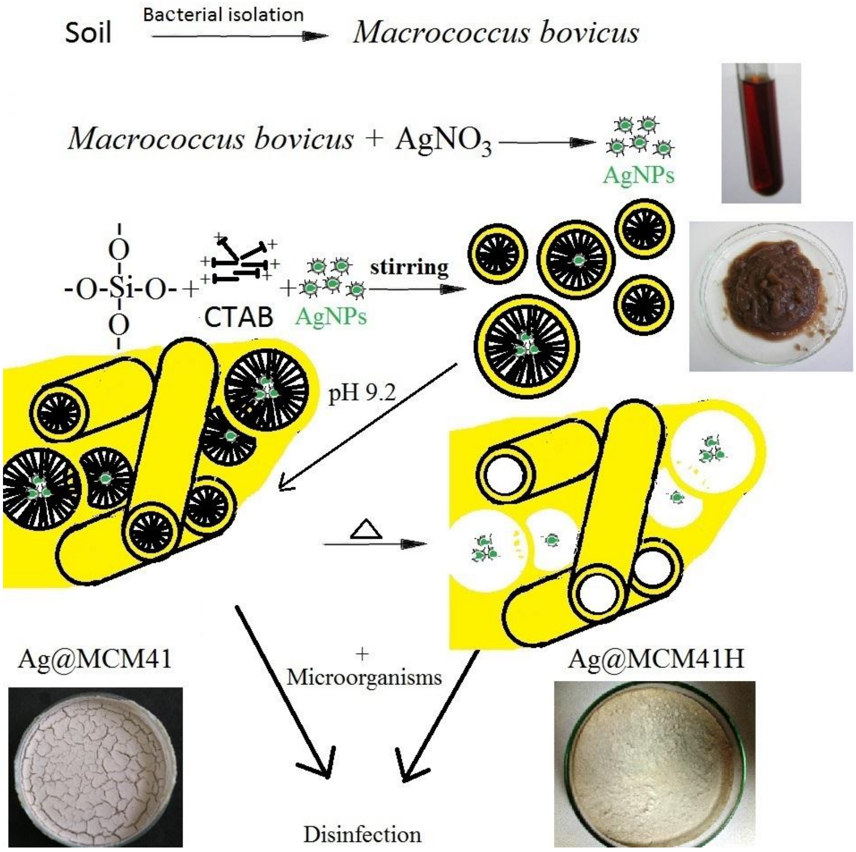

S4. Schematic representation of the synthetic procedure of the studied samples and the distribution of AgNPs inside MCM-41-based composites.

Table S1. Comparison of the antimicrobial activity of AgNPs-modified samples with other published results in literature

Samples	Inhibition zone diameter (mm)			References
	Staphylococcus aureus	*Escherichia coli*	*Candida albicans*	
**AgNPs-MMT**	12	14	12	[S4]
**AgNPs-CTAB-MMT**	14	13	16	[S4]
**S-, NH_2- AgNPs-MMT**	9.8	10.8	-	[S5]
**Ag_2CO_3-MCM-41**		8.3 ± 0.2	-	[S2]
**AgNPs-zeolite**	7.40 ±0.05	7.87 ±0.22	-	[S6]
**AgNPs-gum ghatti**	12.25	8.0–9.0	-	[S7]
**AgNPs–alginate composite on cotton**	3	2	-	[S8]
**Ag1@MCM-41**	12	12	11	Present work
**Ag2@MCM-41**	13	15	12	Present work
**Ag3@MCM-41**	18	19	17	Present work

References

S1. S. Chaliha, K. G. Bhattacharyya, *Catal. Today* **2009**, *141*, 225.

S2. S. Sohrabnezhad, A. Sadeghi, *Appl. Clay Sci.* **2015**, *105*, 217.

S3. M. Sastry, A. Ahmad, I. M. Khan, R. Kumar, Curr. Sci. 2003, 85, 162.

S4. M. S. Abdel-Aziz, K. S. Abou-El-Sherbini, E. M. Hamzawy, M. H. Amr, S. El-Dafrawy, *Appl. Biochem. Biotechnol.* **2015,**176, 2225.

S5. T. Li, O. Lin, Z. Lu, L. He, X. Wang, *Appl. Surf. Sci.* **2014**, 305, 386.

S6. K. Shameli, M. B. Ahmad, M. Zargar, W. M. Z. W. Yunus, N. A. Ibrahim, *Int. J. Nanomed.* **2011**, 6, 331.

S7. A. J. Kora, S. R. Beedu, A. Jayaraman, *Org. Med. Chem. Lett.* **2012**, 2, 17.

S8. M. Zahran, H. B. Ahmed, M. El-Rafie, *Carbohydr. Polym.* **2014**, 108, 145.

Graphical Abstract

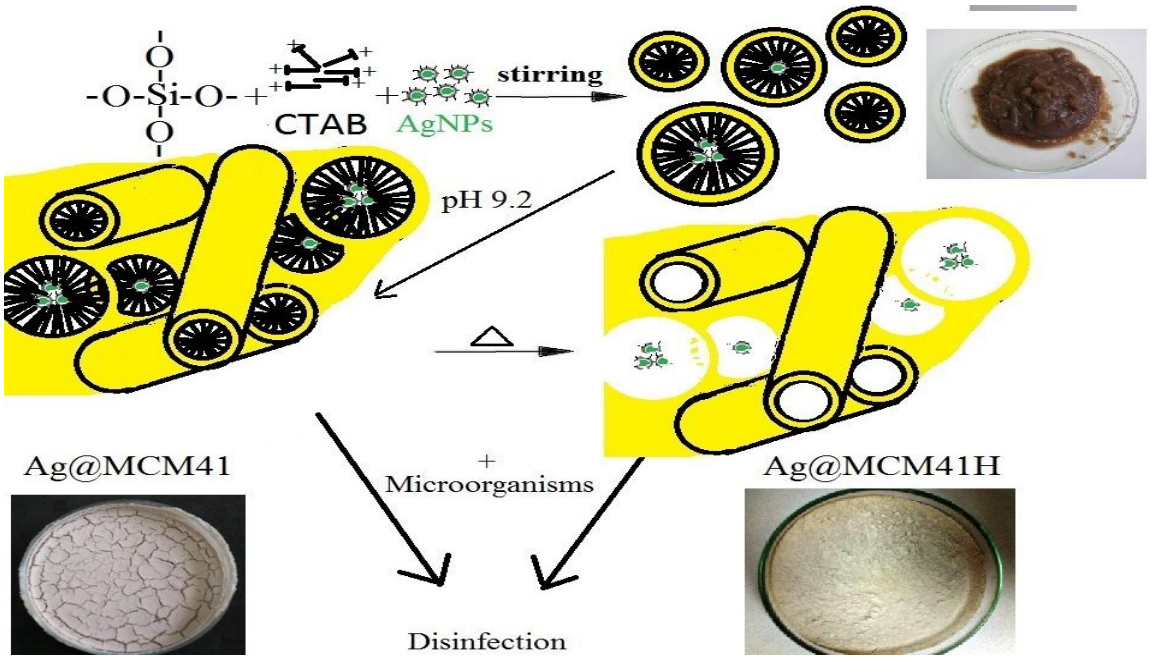

Supplement: Supplementary file 1 — Supplementary [file GCH2-2-1800048-s001.pdf]
